# Placental expression and activity of system A and system L amino acid transporters in pregnancies complicated by diabetes mellitus and fetal growth disorders: a systematic review

**DOI:** 10.3389/fendo.2026.1883051

**Published:** 2026-07-13

**Authors:** Franciszek Ługowski, Julia Babińska, Artur Ludwin, Paweł Jan Stanirowski

**Affiliations:** 1 1^st^ Department of Obstetrics and Gynecology, Medical University of Warsaw, Warsaw, Poland; 2 Doctoral School, Medical University of Warsaw, Warsaw, Poland

**Keywords:** amino acid transporter, diabetes mellitus, fetal growth restriction, fetal macrosomia, placenta

## Abstract

Fetal growth disorders, encompassing both fetal growth restriction (FGR) and fetal macrosomia, are associated with increased neonatal morbidity and mortality rates as well as predispose to metabolic and cardiovascular diseases later in life. Regardless of these negative consequences, the molecular mechanisms underlying different fetal growth patterns remain incompletely understood. Recognizing that fetal growth *in utero* relies on the transplacental transfer of nutrients such as amino acids, we conducted the first systematic review to evaluate the expression and functional activity of System A (SNAT) and System L (LAT) transporter proteins in placentas from pregnancies complicated by diabetes mellitus (DM) and the most common types of fetal growth abnormalities. In alignment with PRISMA guidelines, seventeen studies were selected for data extraction and inclusion within this systematic review. The conducted analysis revealed a distinct functional dichotomy, wherein pregnancies with FGR or small-for-gestational age (SGA) fetuses are characterized by a significant downregulation of System A and L activity and reduced expression of placental isoforms (SNAT1, SNAT2, LAT1). Conversely, pregnancies complicated by DM or fetal overgrowth exhibit upregulated transport capacity, often driven by greater placental mass and increased expression of SNAT1 and LAT1 isoforms, which correlate directly with the neonatal birth weight and adiposity. Alongside the observed differences between the two major cohorts, we documented time-dependent variations within a single pathology; notably, the downregulation of SNAT4 transporter being specific to early-onset FGR, and distinguishing it from the late-onset variant. Our findings demonstrate that during the course of pregnancy, placental amino acid transport capacity adapts to the maternal metabolic environment. As a consequence, the observed alterations in the expression and activity of amino acid transporters at the maternal-fetal interface may determine fetal growth trajectory and body composition, thereby emphasizing the significance of transplacental nutrient flux as a critical target for investigating the developmental origins of metabolic health in the offspring.

**Systematic review registration:**

https://www.crd.york.ac.uk/PROSPERO/, identifier CRD420251208578.

## 1 Introduction

Optimal intrauterine fetal growth plays a pivotal role not only in determining pregnancy outcomes but also in shaping an individual’s long-term health. During the perinatal period, the most extreme deviations from the normal fetal growth trajectory, such as fetal growth restriction (FGR) and fetal macrosomia, are associated with increased risks of neonatal morbidity and mortality (1, 2). Moreover, these growth abnormalities are strongly correlated with an increased susceptibility to metabolic and cardiovascular diseases during childhood and adolescence (3–5).

The efficient transport of nutrients from the maternal to the fetal circulation constitutes one of the key determinants of fetal growth *in utero* (6–8). The placenta, specifically the syncytiotrophoblast layer, plays a central role in this process, serving as the primary regulator of the nutrient flux between the mother and fetus. Among the numerous factors influencing placental nutrient transfer, such as uteroplacental blood flow, substrate concentration gradients, and placental metabolism, the expression and activity of specific transporter proteins located in syncytial membranes are of particular importance (9).

**Figure 1:**
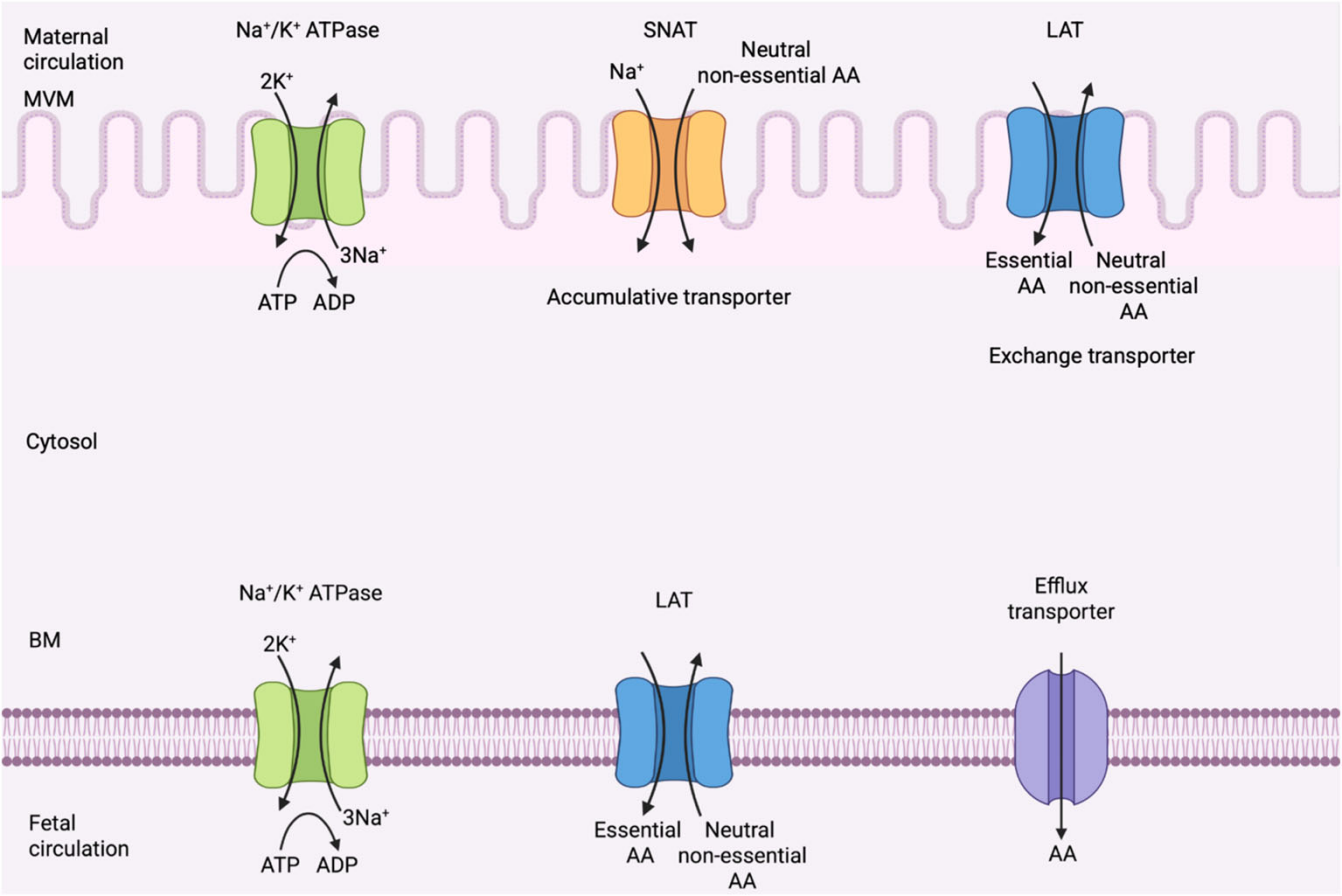
System A and system L amino acid transport in human syncytiotrophoblast. Created with biorender.com. AA, amino acid; ADP, adenosine diphosphate; ATP, adenosine triphosphate; BM, basal membrane; LAT, L-type amino acid transporter; MVM, microvillous membrane; SNAT, sodium-coupled neutral amino acid transporter.

Besides glucose and lipids, amino acids are essential for proper fetal development due to their contribution to protein synthesis, energy metabolism, and cellular signaling (10). Of the various systems facilitating maternal-fetal amino acid transfer, System A and System L represent the most thoroughly investigated so far (6, 11–13). The former is responsible for the sodium-dependent uptake of non-essential neutral amino acids and includes isoforms such as SNAT1, SNAT2, SNAT4, and SNAT5 (13). On the contrary, System L, comprising LAT1 and LAT2 transporters, represents sodium-independent exchangers that mediate the cellular uptake of essential amino acids, such as branched-chain (L-leucine) and neutral aromatic amino acids (L-phenylalanine) (14, 15),. Beyond differences in substrate affinity, these two systems exhibit distinct spatial distributions within the placental syncytium; specifically, LAT is localized to both the microvillous (MVM) and basal membranes (BM), whereas SNAT is predominantly situated in the MVM (13). **Figure 1** depicts the localization and mechanisms of action of SNAT and LAT transporters in the human placenta.

Evidence derived from animal studies, including trophoblast-specific SNAT2/SNAT4 gene knockout mice and nutrient-restricted baboon models, indicates that reduced placental System A and System L transport activity may contribute to placental hypoplasia and FGR (16, 17). In humans, it has been demonstrated that the concentrations of amino acids in the blood of neonates diagnosed with FGR are significantly lower compared to appropriately grown fetuses, and placental activity of certain neutral amino acid transporters - including those for alanine, serine, and glycine - is markedly reduced among small for gestational age (SGA) infants (18–20). Although these findings suggest that impaired transplacental amino acid transport may represent a mechanism contributing to abnormal fetal growth, the causal relationship between alterations in placental transport capacity and the development of FGR is still to be determined.

Unlike gestations characterized by restricted fetal growth, pregnancies involving diabetes mellitus (DM) often lead to fetal macrosomia and birth of large for gestational age (LGA) neonates (21, 22). Emerging data suggest that fetal overgrowth in DM-complicated pregnancies, similarly to glucose and lipids, may be partly mediated by upregulated amino acid transport in the placenta (23, 24).

To date, no systematic review on the expression and activity of system A and L amino acid transporters in the human placenta has been published. Accordingly, the objective of the present study is to fill the existing research gap by systematically evaluating the available evidence on the expression levels and functional activity of these transport proteins in pregnancies affected by DM and the most common types of fetal growth disorders.

## 2 Materials and methods

### 2.1 Search strategy and study selection

The systematic review was conducted in accordance with the Preferred Reporting Items for Systematic Reviews and Meta-Analyses (PRISMA) guidelines (25), and the study protocol has been registered in the PROSPERO database with the registration number CRD420251208578.

A literature search was performed in PubMed, Embase, Scopus, and Web of Science databases from 11 November 2025 to 31 December 2025. The search strategy combined controlled vocabulary and free-text terms related to amino acid transporter proteins in the placenta (“system A,” “system L,” “SLC38A,” “SLC7A,” “LAT,” “SNAT”) and pregnancy outcomes (“fetal growth restriction,” “small for gestational age,” “fetal macrosomia,” “large for gestational age,” “diabetes mellitus,” “type 1 diabetes,” “type 2 diabetes,” “gestational diabetes mellitus”). The full search syntax for each database is provided in **Supplementary File S1**.

After deduplication, two reviewers (F.Ł. and J.B.) independently screened titles and abstracts, followed by full-text review of eligible studies. Reference lists of all included papers and relevant reviews were screened for additional studies. No language or time restrictions were applied. Discrepancies were resolved by consensus or by consultation with the senior author (P.J.S.).

### 2.2 Eligibility criteria

Studies were included if they:

1. Investigated the expression or activity of system A or system L amino acid transporter proteins in the human placenta;
2. Reported data from pregnancies complicated by FGR, SGA, LGA, fetal macrosomia, type 1 diabetes mellitus (T1DM)/type 2 diabetes mellitus (T2DM)/gestational diabetes mellitus (GDM); and
3. Included an appropriate control group of healthy pregnancies.

Studies limited to animal models, *in vitro* cell lines without direct human placental tissue analysis, mRNA expression analysis, case reports, reviews, and conference abstracts without full data availability were excluded.

### 2.3 Data extraction and quality assessment

Two reviewers (F.Ł. and J.B.) independently extracted data using a prepiloted form, recording study design, population characteristics, diagnostic criteria for FGR/SGA/LGA/fetal macrosomia, type of diabetes, gestational age at sampling or delivery, methods used to assess transporter expression or activity, and principal quantitative outcomes. When multiple transporter isoforms were reported, data were extracted separately for each.

Study quality and risk of bias were evaluated using the Risk of Bias in Non-randomized Studies of Exposure (ROBINS-E) tool (26). The ROBINS-E tool evaluates seven domains of potential bias: confounding, selection of participants, classification of exposures, deviations from intended exposures, missing data, measurement of outcomes, and selection of the reported result. Each study was judged as having low risk, some concerns, high risk, or very high risk of bias in each domain, with an overall judgment derived accordingly. Discrepancies between reviewers were resolved by discussion or arbitration with the senior author (P.J.S.).

### 2.4 Data synthesis

Given expected heterogeneity in study design, population characteristics, and outcome measures, results were synthesized descriptively. Expression or activity changes in system A and system L transporters were summarized relative to matched controls, stratified by pathological condition (FGR/SGA, LGA/fetal macrosomia, and diabetes subtypes).

## 3 Results

### 3.1 Search results and study selection

**Figure 2:**
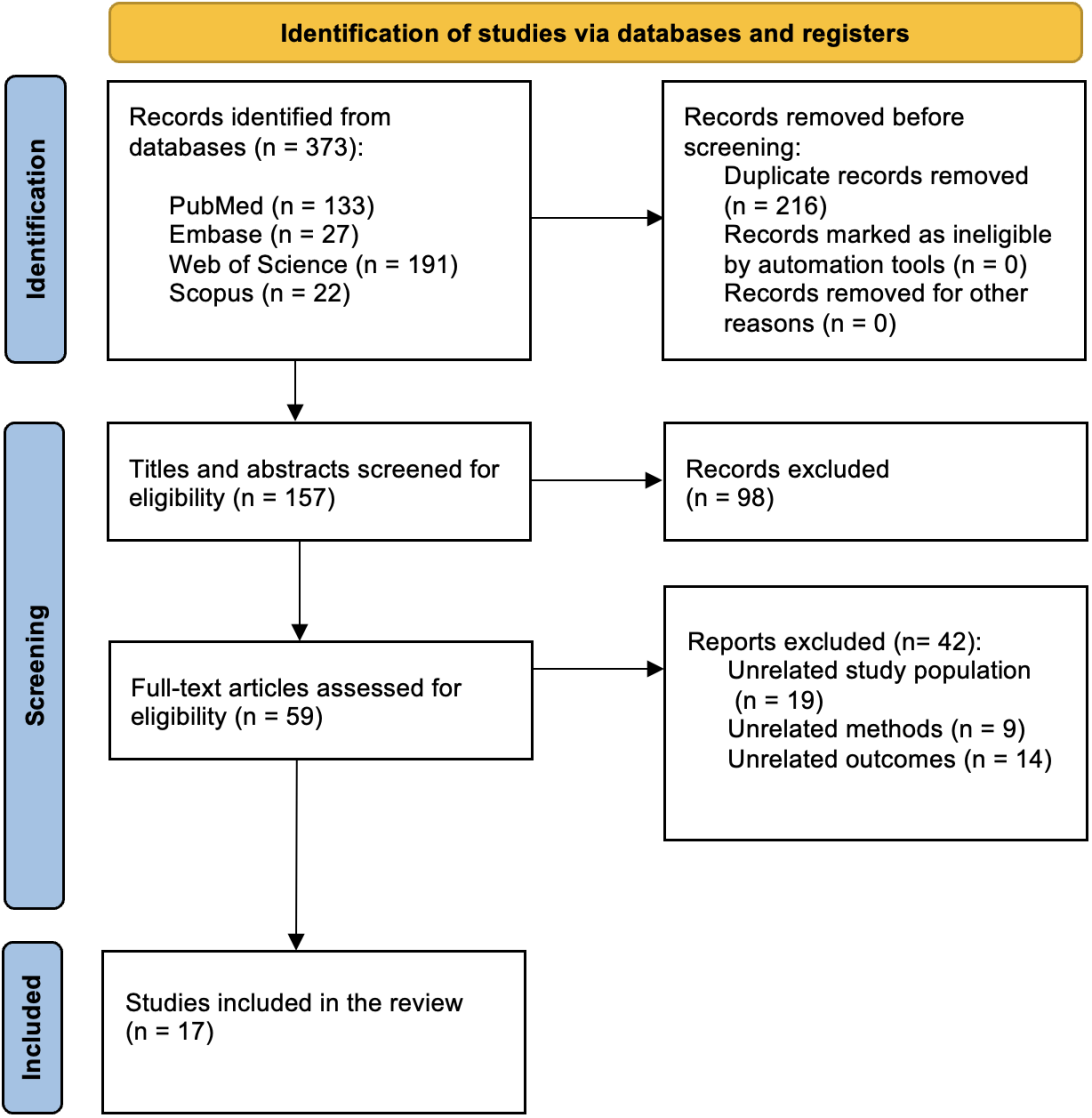
PRISMA flow chart of the screening process.

**Figure 3:**
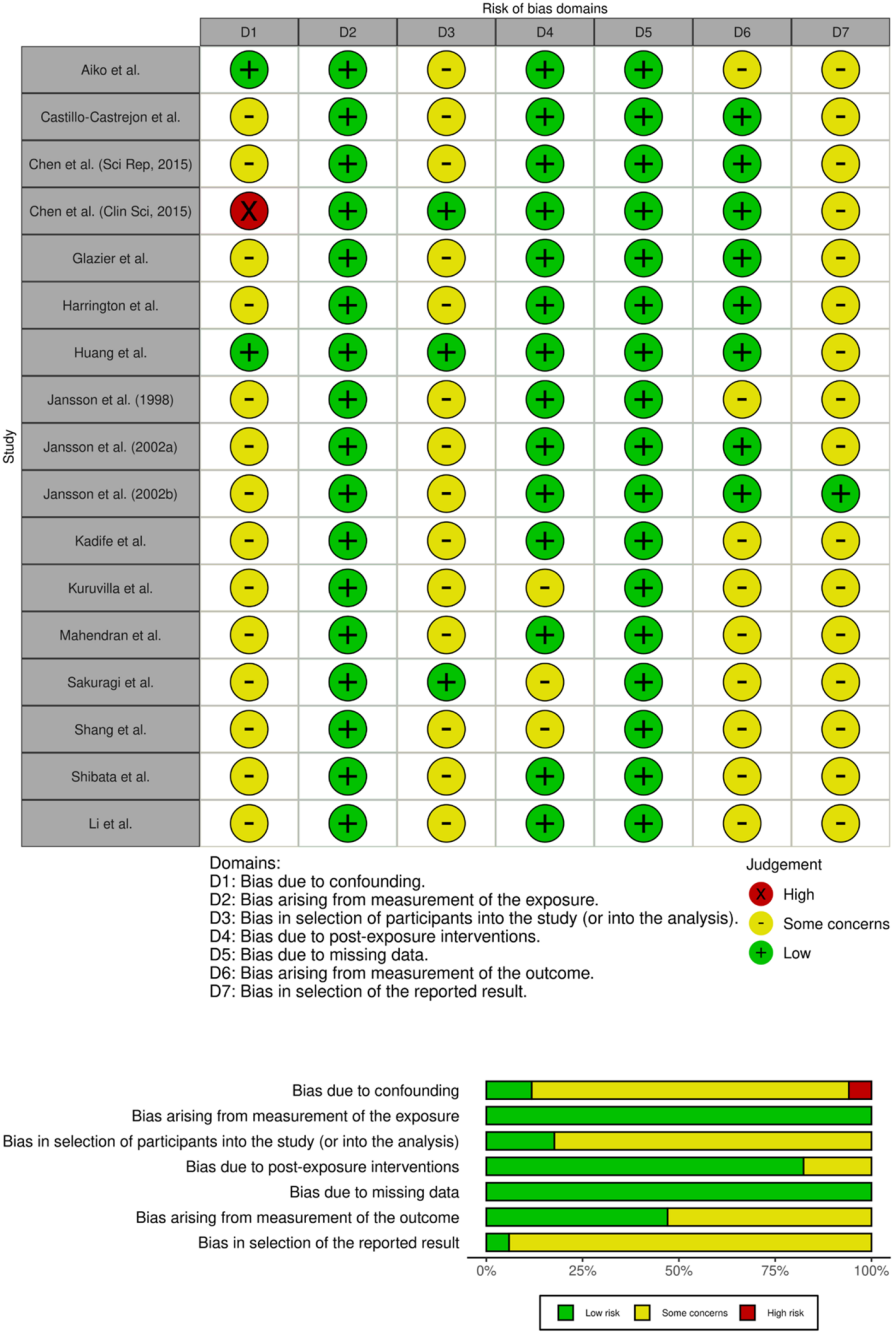
Assessment of the study bias according to the ROBINS-E tool. Risk of bias judgments are interpreted as follows: Low risk: The study is comparable to a well-performed randomized trial with regard to this domain; Some concerns: The study provides sound evidence but has minor methodological limitations; High risk: The study has serious methodological flaws that weaken confidence in the results; Very high risk: The study has fundamental flaws making the findings highly unreliable.

**Figure 4:**
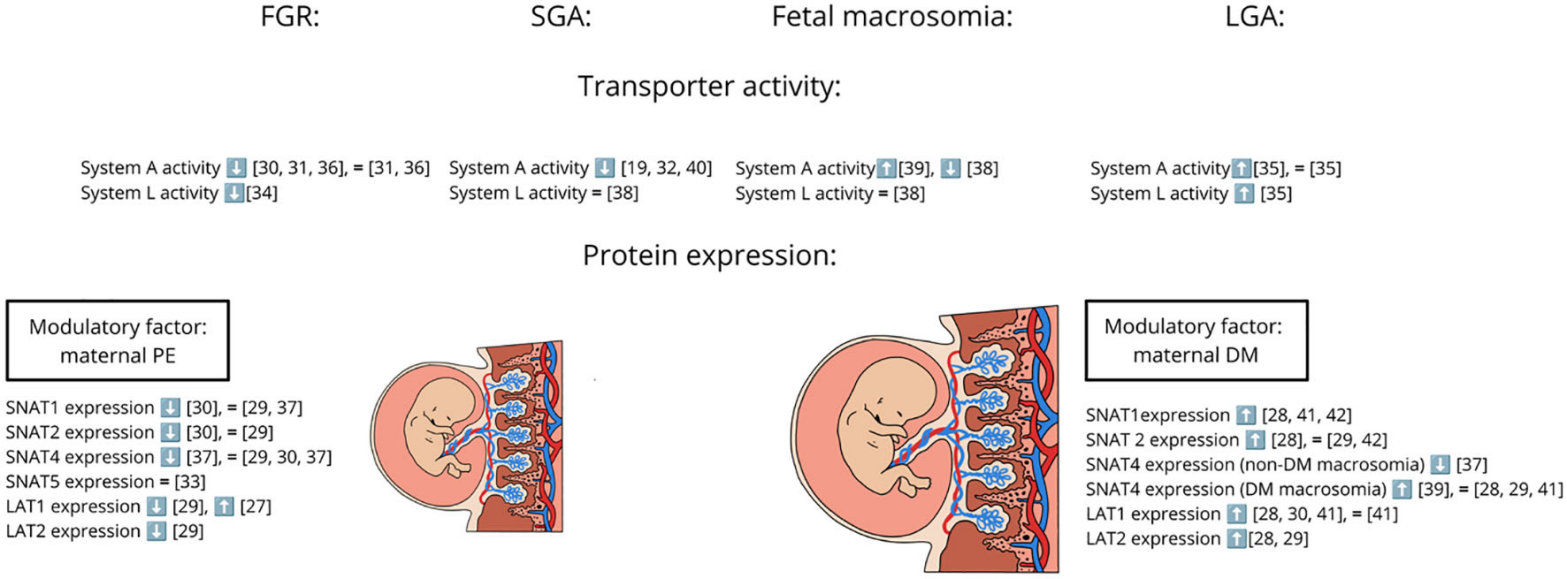
Summary of placental amino acid transport alterations in pregnancies complicated by fetal growth disorders. DM, diabetes mellitus; FGR, fetal growth restriction; LAT1, L-type amino acid transporter 1; LAT2, L-type amino acid transporter 2; SGA, small for gestational age; LGA, large for gestational age; PE, preeclampsia; SNAT1, sodium-coupled neutral amino acid transporter 1; SNAT2, sodium-coupled neutral amino acid transporter 2; SNAT4, sodium-coupled neutral amino acid transporter 4; SNAT5, sodium-coupled neutral amino acid transporter 5;.

A systematic literature search retrieved 373 articles for initial analysis (**Figure 2**). Following the removal of 216 duplicates, 157 titles and abstracts were screened for eligibility. A total of 59 publications underwent an in-depth full-text analysis, resulting in 42 studies being excluded from further assessment. Ultimately, 17 studies met the inclusion criteria and were incorporated into this systematic review (**Table 1**). **Figure 3** illustrates the evaluation of potential bias within each included study, as determined by the ROBINS-E tool. **Figure 4** represents a visual summary of placental amino acid transport alterations in pregnancies complicated by fetal growth disorders.

**Table 1:** Characteristics of studies included in the analysis.

Author	Year	Number of patients	Pathological condition	Methodology	Amino-acid transporters	Results
Aiko et al. (27)	2014	n = 40 (FGR, n = 10; PE, n = 10, full-term control, n = 10; pre-term control, n = 10)	FGR	Protein expression: IHC of formalin-fixed, paraffin-embedded placental tissue + image analysis software	SLC7A5 (LAT1)	1. LAT1 expression levels were significantly higher in the syncytiotrophoblast of FGR placentas compared to both full-term and pre-term controls (p < 0.05).
Castillo-Castrejon et al. (28)	2021	n = 18 (T2DM, n = 9; NGT, n = 9)	T2DM	Protein expression: WB in isolated MVM and BM fractions System A transporter activity: Na^+^-dependent 14C-MeAIB uptake in isolated MVM vesicles	SLC38A1 (SNAT1), SLC38A2 (SNAT2), SLC38A4 (SNAT4), SLC7A5 (LAT1), SLC7A8 (LAT2)	1. SNAT1 and SNAT2 expression in the MVM was significantly increased among T2DM women (+26%, p=0.05, +47%, p=0.01, respectively) when compared to NGT. 2. No differences in SNAT4 expression in the MVM between both groups were noted. 3. LAT1 and LAT2 expression in the MVM was significantly increased in T2DM women (+27%, p=0.01; +59%, p=0.01, respectively) when compared to NGT. No differences in transporters expression in BM were noted. 4. SNAT1 expression in the MVM of T2DM women was positively associated with FBW (r = 0.84, p = 0.004), but not with neonatal fat mass. 5. SNAT2 expression in the MVM was not correlated with FBW or fat mass in neonates born to T2DM women. 6. SNAT4 expression in the MVM was negatively correlated with FBW in NGT (r =− 0.72, p = 0.02) and T2DM women (r = − 0.71, p = 0.01), but not with neonatal adiposity. 7. LAT1 expression in the T2DM group correlated with FBW (r = 0.59, p = 0.04) and neonatal fat mass (r = 0.76, p = 0.06). 8. LAT2 expression was not correlated with FBW nor neonatal fat mass.
Chen et al. (29)	2015	n = 48 (SGA, n = 16; LGA, n = 14; AGA, n = 17)	SGA, LGA	Protein expression: WB in total placental tissue	SLC38A1 (SNAT1), SLC38A2 (SNAT2), SLC38A4 (SNAT4), SLC7A5 (LAT1), SLC7A8 (LAT2)	1. Protein expression of LAT1 and LAT2 was significantly lower in the SGA group compared to the AGA and LGA groups (p < 0.01). 2. LAT 1 and LAT 2 expression was positively correlated with the FBW in total study population (p < 0.01). 3. The expression levels of System A transporters (SNAT1, SNAT2, and SNAT4) did not differ significantly between the groups.
Chen et al. (30)	2015	n = 44 (pre-term FGR, n = 14; term FGR, n = 11; preterm control, n = 12; term control, n = 7)	FGR	Protein expression: WB in total placental homogenates and isolated MVM fractions System A transporter activity: 14C-MeAIB uptake in isolated MVM vesicles using rapid filtration	System A, SLC38A1 (SNAT1), SLC38A2 (SNAT2)	1. Protein expression of SNAT-1 and SNAT-2 was significantly decreased in the MVM of FGR placentas (–42%, p < 0.05; –31%, p < 0.05, respectively). 2. No significant change could be observed in the expression of the SNAT-4 isoform. 3. MVM System A activity was significantly lower in the FGR cohort (76.5 ± 14.9 pmol/mg × 30 s) compared to the control group (271.1 ± 33.3 pmol/mg × 30 s), representing a 72% reduction (p < 0.0001). 4. The regression analysis indicated a strong positive correlation between gestational age and System A activity within the FGR group (r = 0.79, p < 0.001).
Glazier et al. (31)	1997	n = 26 (FGR, n = 16, AGA, n = 10)	FGR	System A transporter activity: radiolabeled substrate uptake assays in isolated MVM vesicles	System A (SLC38 family)	1. In the FGR group System A activity in MVM vesicles was decreased compared to AGA controls (0.026 ± 0.004 vs. 0.053 ± 0.005 nmol/mg protein/30 s; p < 0.001). 2. System A activity showed severity-dependent impairment: levels were preserved in mild FGR but significantly reduced in severe phenotypes.
Harrington et al. (32)	1999	n = 49 (SGA, n = 25; AGA, n = 24)	SGA	System A transporter activity: radiolabeled substrate uptake assays in isolated MVM vesicles	System A (SLC38 family)	1. System A activity was significantly lower in MVM vesicles from SGA placentas compared to AGA (p = 0.006). 2. In the SGA group System A activity correlated positively with FBW, neonatal subscapular and triceps skin-fold thickness (r = 0.48, p < 0.05; r = 0.42, p < 0.05, respectively), and PW (r = 0.42, p < 0.05).
Huang et al. (33)	2018	n = 43 (FGR, n = 8; PE, n = 5; PE + FGR, n = 10 and controls - term, n = 13; preterm, n = 7)	FGR	Protein expression: WB in total tissue lysates and isolated membrane fractions	SLC7A7 (y+LAT1), SLC38A5 (SNAT5)	1. The expression of SNAT5 in FGR (+/- PE) placentas was unaltered. 2. y+LAT1 transporter was found to be significantly down-regulated in FGR placentas (p < 0.05). 3. y+LAT1 transporter was significantly up-regulated in PE+FGR and PE placentas (p < 0.05).
Jansson et al. (34)	1998	n = 14 (FGR, n = 8; control, n = 6)	FGR	System L transporter activity: radiolabeled substrate uptake assays in isolated MVM and BM vesicles	System L (SLC38 family)	1. System L activity was significantly reduced in FGR, in both the syncytial MVM (-46%, p < 0.05) and BM (-38%, p < 0.05).
Jansson et al. (35)	2002a	n = 84 (T1DM, n = 8; T1DM/LGA, n = 13; GDM, n = 10; GDM/LGA, n = 10; LGA, n = 8; NGT, n = 35)	T1DM, GDM, LGA	System A and System L transporter activity: radiolabeled substrates (14C-MeAIB and 3H-leucine) uptake in isolated MVM and BM fractions and rapid filtration techniques	System A (SLC38 family) and System L (SLC7 family)	1. System A activity in MVM was significantly increased (65–80%, p < 0.05) in all groups with diabetes independent of fetal overgrowth. 2. MVM System A activity was unaffected in placentas of non-diabetic pregnancies with LGA fetuses and controls. 3. System L activity was increased in GDM women delivering LGA neonates (p < 0.05). 4. No differences in the activity of System A and System L in the syncytial BM were observed.
Jansson et al. (36)	2002b	n = 36 (preterm FGR, n = 8; term FGR, n = 11; preterm controls, = 8; term controls, n = 9)	FGR	System A transporter activity: Na+-dependent uptake of 14C-MeAIB in isolated MVM and BM vesicles	System A (SLC38 family)	1. System A activity in MVM was reduced by 50% in the preterm FGR cohort in comparison with the preterm controls (p < 0.05). 2. No significant change for MVM System A activity was reported in the term FGR group compared to gestational age-matched controls. 3. MVM System A activity was positively correlated with gestational age in both FGR cohorts (r = 0.74, p < 0.01). No such association was reported for the controls. 4. System A activity in BM was not altered in FGR cohorts.
Kadife et al. (37)	2022	n = 119 (early preterm <34 weeks of gestation: PE + FGR, n = 22; FGR, n = 13; preterm controls, n = 20 and late preterm 34–36 weeks: PE + FGR, n = 11; FGR, n = 29; preterm controls, n = 24)	FGR	Protein expression: WB in total placental tissue	SLC38A1 (SNAT1), SLC38A4 (SNAT4)	1. Significantly lower SNAT4 expression was observed in early preterm FGR (+/- PE) as compared to controls (p < 0.01). 2. SNAT4 expression remained unaltered in late preterm cohorts. 3. SNAT1 levels showed no significant differences across early and late preterm cohorts.
Kuruvilla et al. (38)	1994	n = 43 (DM with AGA, n = 12; DM with fetal macrosomia, n = 13; controls without fetal macrosomia, n = 18)	T1DM, GDM, fetal macrosomia	System A and System L transporter activity: Na^+^-dependent 14C-MeAIB and 14C-leucine uptake in isolated MVM vesicles	System A (SLC38 family) and System L (SLC7 family)	1. System A transporter activity was 49% lower in DM –associated fetal macrosomia compared to non-diabetic controls (p < 0.02). 2. No significant difference in System A activity between DM-AGA and control cohorts was noted. 3. No significant difference regarding the activity of system L between the subgroups was observed.
Li et al. (39)	2011	n = 52 (fetal macrosomia >4000g; n = 20; low birth weight <2500g, n = 22; normal birth weight 2500-4000g, n = 10)	fetal macrosomia	Protein expression: WB in total placental tissue System A transporter activity: isotope incorporation assay measuring 3H-proline uptake in placental villous fragments	System A (SLC38 family), SLC38A4 (SNAT4)	1. SNAT4 expression in the fetal macrosomia group was significantly higher compared to the normal birth weight cohort (p < 0.05). 2. System A activity in the macrosomia group was increased (1.2 ± 0.20 vs. 1.0 ± 0.18; p < 0.05), compared to the normal birth weight cohort.
Mahendran et al. (40)	1993	n = 32 (SGA, n = 14; AGA controls, n = 18)	SGA	System A transporter activity: Na^+^-dependent 14C-MeAIB uptake in isolated MVM vesicles	System A (SLC38 family)	1. System A activity was significantly lower (63% reduction) in SGA vesicles (p < 0.001). 2. Vmax of the amino acid transfer was significantly reduced (p < 0.001), with no change in transporter affinity (Km).
Sakuragi et al. (41)	2024	n =101 (GI, n = 61; non-GI, n = 40)	T2DM, GDM, overt DM, LGA	Protein expression: IHC of formalin-fixed, paraffin-embedded placental tissue + image analysis software	SLC38A1 (SNAT1), SLC7A5 (LAT1)	1. The per villus unit expression (density) level of SNAT1 was significantly lower in the GI group than in the non-GI group [0.59 (0.48-0.80) vs. 0.76 (0.62-0.81), p= 0.003. 2. No statistically significant difference for LAT1 was found [0.55 (0.51-0.61 vs. 0.58 (0.54-0.62), p = 0.20). 3. The per placental unit (total) expression levels of both SNAT1 and LAT1 were significantly higher in the GI group compared to the non-GI cohort (both p < 0.001). 4. SNAT1 and LAT1 expression was significantly increased in the GI/LGA group compared to the non-GI/non-LGA cohort (both p < 0.001).
Shang et al. (42)	2018	n = 50 (GDM, n = 50, including: fetal macrosomia, n = 23; normal weight infants, n = 27)	GDM, macrosomia	Protein expression: WB in total placental tissue	SLC38A1 (SNAT1), SLC38A2 (SNAT2), SLC38A4 (SNAT4)	1. Placental expression of SNAT1 was significantly higher in fetal macrosomia compared to normal weight controls (0.29 ± 0.11, p < 0.05). No differences for SNAT2 (0.34 ± 0.15 vs. 0.30 ± 0.19) and SNAT4 expression (0.29 ± 0.09 vs. 0.31 + 0.16) were found. 2. The expression of SNAT1 was positively correlated with FBW (p < 0.05).
Shibata et al. (19)	2008	n = 29 (SGA, n = 5; PE + SGA, n = 5; PE, n = 10; controls, n = 9)	SGA	System A transporter activity: Na^+^-dependent 14C-MeAIB uptake in isolated placental villous tissue	System A (SLC38 family)	1. System A activity was significantly reduced in SGA placentas without PE compared to normal controls (p < 0.001). 2. System A activity was unaltered in pregnancies with PE + SGA.

AGA, appropriate for gestational age; BM, basal membrane; DM, diabetes mellitus; FBW, fetal birth weight; FGR, fetal growth restriction; GDM, gestational diabetes mellitus; GI, glucose intolerance; IHC, immunohistochemistry; LAT1, L-type amino acid transporter 1; LAT2, L-type amino acid transporter 2; LGA, large for gestational age; MeAIB - [11C] methyl alpha-aminoisobutyric acid; MVM, microvillous membrane; NGT, normoglycemic controls; PE, preeclampsia; PW, placental weight; SGA, small for gestational age; SLC, solute carrier; SNAT1, sodium-coupled neutral amino acid transporter 1; SNAT2, sodium-coupled neutral amino acid transporter 2; SNAT4, sodium-coupled neutral amino acid transporter 4; SNAT5, sodium-coupled neutral amino acid transporter 5; T1DM, type 1 diabetes mellitus; T2DM, type 2 diabetes mellitus; y^+^LAT1, y+ L-type amino acid transporter; WB, Western blot.

### 3.2 Expression of system A transporters in fetal growth restriction and small-for-gestational-age infants

The expression profiles of System A amino acid transporters were scrutinized in three studies concerning FGR, which collectively documented a general tendency toward decreased or unaltered SNAT protein levels in the placental syncytiotrophoblast (30, 33, 37). Research conducted by Chen et al. demonstrated that, in comparison to control placentas, those affected by FGR exhibited a 42% decline in SNAT1 and a 31% decline in SNAT2 expression within the microvillous membrane (p < 0.05) (30). In addition, no significant change in SNAT4 levels was observed. Conversely, in a more recent study by Kadife et al., no statistically significant differences in SNAT1 expression were found in early and late preterm cases of FGR, whereas SNAT4 levels decreased in placentas of growth-restricted fetuses born before 34 weeks of gestation (37). Finally, Huang et al. reported no significant differences in SNAT5 expression (33).

In an investigation into SGA infants, Chen et al. analyzed the placental expression of SNAT1, SNAT2, and SNAT4 amino acid transporters, concluding that no significant discrepancies existed relative to appropriate for gestational age (AGA) and LGA cohorts (29).

In two studies evaluating the potential impact of maternal preeclampsia (PE) on transporter expression, Huang et al. reported unaltered SNAT5 levels in pregnancies with FGR +/- PE, whereas Kadife et al. observed a reduction in SNAT4 expression in pre-term FGR +/- PE placental tissues (19, 33, 37).

### 3.3 Activity of system A transporters in fetal growth restriction and small-for-gestational-age infants

The functionality of System A in pregnancies with FGR was evaluated in two studies, both demonstrating a significant reduction in transport efficiency (30, 31). In the study by Glazier et al., transport activity in the MVM vesicles from the AGA group was significantly higher than that observed in the FGR group (0.053 ± 0.005 vs. 0.026 ± 0.004 nmol/mg protein/30 s; p < 0.001) (31). Interestingly, when stratified by clinical severity, System A activity in the mildest FGR phenotype was not significantly altered as compared to controls, whereas it remained significantly reduced in the more severe subgroups, indicating a severity-dependent impairment of amino acid transport. Evidence for this phenotype-specific dysfunction is further reinforced by the findings of Jansson et al., who observed a 50% reduction in MVM System A activity exclusively in preterm FGR cohorts (p < 0.05), whereas activity in term FGR placentas remained comparable to gestational age-matched controls (36). In accordance with the two abovementioned studies, Chen et al., described a significantly lower transport activity in the FGR cohort (76.5 ± 14.9 pmol/mg × 30 s) compared to the control group (271.1 ± 33.3 pmol/mg × 30 s), representing a 72% reduction (p < 0.0001) (30). In addition, both Chen et al. and Jansson et al. documented a strong positive correlation between gestational age and transporter activity, specifically within the FGR group (r = 0.79 and r = 0.74, respectively; both p < 0.01), a relationship that was absent in the control population (30, 36). Concerning the spatial distribution of transporters, Jansson et al. noted that System A activity in the BM was not significantly altered in FGR, suggesting this functional deficit is confined solely to the maternal-facing membrane of the syncytiotrophoblast (36).

Evidence from three distinct reports regarding System A kinetics in SGA-affected pregnancies demonstrates a consensus on a substantial decline in transport activity (19, 32, 40). Harrington et al. reported significantly reduced System A activity in syncytiotrophoblast microvillous vesicles from SGA-affected pregnancies in comparison with the AGA control group (0.027 ± 0.004 vs. 0.045 ± 0.005 nmol/mg vesicle protein/30 s, p = 0.006). Moreover, in the former cohort, transport activity remained positively correlated with several neonatal anthropometric and placental indices, including subscapular skinfold thickness (r = 0.48, p < 0.05), triceps skinfold thickness (r = 0.42, p < 0.05), and placental weight (r = 0.42, p < 0.05) (32). Based on the study by Mahendran et al., the initial rate of System A amino acid transporter activity, assessed via Na^+^-dependent methylaminoisobutyric acid (MeAIB) uptake, was significantly lower (by 63%) in MVM vesicles from SGA placentas compared to AGA controls (0.014 ± 0.002 vs. 0.038 ± 0.005 nmol/mg protein; p < 0.001) (40). Additional kinetic analysis revealed that the maximal transport velocity (V_max_) was significantly reduced in the SGA group (0.24 ± 0.03 nmol/mg protein/30 s vs. 0.64 ± 0.09 nmol/mg protein/30 s; p < 0.001), whereas there was no difference in the affinity (K_m_) of the transporters (2.10 ± 0.83 mM for SGA vs. 5.35 ± 1.61 mM for AGA; p = 0.09) (40). Lastly, in the study by Shibata et al., the authors confirmed significantly reduced system A activity in SGA placentas from women without PE compared to healthy controls (p < 0.001) (19). Contrarily, in the case of coexistence of maternal PE and SGA, transport activity remained unaltered.

### 3.4 Expression of system A transporters in fetal macrosomia and large-for-gestational-age infants

Fetal macrosomia, not associated with maternal obesity/DM, constitutes a rare entity; therefore, only a single study has evaluated the expression of System A transporters in cases of isolated fetal overgrowth (39). An analysis conducted by Li et al. on SNAT4 expression in non-DM participants revealed that protein levels were significantly higher in fetal macrosomia cases (>4000g) compared to the control group of infants weighing 2500-4000g (p < 0.05) (39). Contrary to these findings, in pregnancies complicated by GDM, Shang et al. reported that placental expression of the SNAT1 isoform was significantly upregulated in cases of fetal macrosomia, compared to normal weight controls (0.29 ± 0.11, p < 0.05), as well as remained positively associated with fetal birth weight (FBW) (p < 0.05) (42). Furthermore, the study found no significant differences in the expression levels of SNAT2 (0.34 ± 0.15 vs. 0.30 ± 0.19) and SNAT4 proteins (0.29 ± 0.09 vs. 0.31 ± 0.16) (42).

With regard to LGA infants, a single study by Chen et al. evaluated the placental expression of SNAT1, SNAT2, and SNAT4 transporters and found no significant differences when compared to AGA and SGA populations (29).

### 3.5 Activity of system A transporters in fetal macrosomia and large-for-gestational-age infants

Similarly to protein expression, only one study assessed the activity of System A in isolated fetal macrosomia (39). In another report authors evaluated this matter in DM-associated fetal overgrowth (38). The two studies reported inconsistent findings (38, 39). First, Kuruvilla et al. demonstrated that System A activity was reduced by 49% in MVM from placentas of macrosomic infants born to T1DM/GDM mothers (p < 0.02) (38). The observed decrease was mainly driven by a significant reduction in the transport maximal velocity (V_max_) in the macrosomia group as compared to controls (0.47 ± 0.07 vs. 0.96 ± 0.13 nmol/mg protein/30 s; p < 0.02), whereas transporter affinity (K_m_) remained generally unaltered (2.10 ± 1.08 vs. 1.44 ± 0.80 mM) (38). Contrary evidence was reported by Li et al., who found that activity of System A in cases of isolated fetal macrosomia >4000g was significantly increased (1.2 ± 0.20 vs. 1.0 ± 0.18; p < 0.05) in comparison with the normal birth weight cohort weighing between 2500 and 4000g (39).

Regarding LGA infants, Jansson et al. observed that System A activity was unaltered in placentas from pregnancies uncomplicated by diabetes in both MVM and BM (35). However, if GDM or T1DM co-occurred with LGA, the System A activity in MVM was significantly increased (p < 0.05) (35).

### 3.6 Expression of system A transporters in diabetes mellitus

The expression levels of System A amino acid transporters across various forms of DM were evaluated in two investigations (28, 41). In the study by Castillo-Castrejon et al., the authors reported that SNAT1 protein expression in T2DM women was increased by 26% (p = 0.05) and remained positively associated with FBW (r = 0.84, p = 0.004) (28). Conversely, Sakuragi and colleagues described significantly lower transporter expression (per villus unit) in the cohort comprising GDM, overt DM, and T2DM women when compared to healthy controls [0.59 (0.48–0.80) vs. 0.76 (0.62–0.81), p = 0.003] (41). Notably, in the same research SNAT1 expression measured per placental unit was increased among glucose intolerant individuals (p < 0.001). Regarding the SNAT2 isoform, it was identified that while transporter expression in the MVM of placentas from the T2DM group exceeded controls by 47% (p = 0.01), it did not correlate with FBW or neonatal fat mass (28). In contrast, the expression of SNAT4 did not differ significantly between the two groups and was negatively correlated with FBW in both T2DM (r = − 0.71, p = 0.01) and healthy control populations (r = − 0.72, p =0.02) (28).

### 3.7 Activity of system A transporters in diabetes mellitus

Data regarding System A activity in diabetic pregnancies were analyzed in two studies and remain conflicting (35, 38). Kuruvilla et al. reported that System A activity was significantly reduced (by 49%) in DM-affected pregnancies with concomitant fetal macrosomia as compared to controls (p < 0.02), a functional impairment driven by decreased maximal velocity (V_max_) rather than altered transporter affinity (38). Conversely, Jansson et al. documented a significant 65–80% increase in MVM System A activity among subjects with T1DM and GDM, which occurred independently of fetal overgrowth (35). No significant differences in the activity of System A were found for syncytial BM (35).

### 3.8 Expression of system L transporters in fetal growth restriction and small-for-gestational-age infants

In FGR-affected pregnancies without concomitant PE, Huang et al. observed a decrease in the expression of the System y^+^LAT (SLC7A7) isoform, y^+^LAT1, whereas a combination of maternal PE + FGR resulted in upregulated protein levels (19, 33, 37). Contrary to these findings, Aiko et al. reported significantly higher levels of LAT1 in isolated FGR placentas compared to both full-term and pre-term controls (p < 0.05) (27).

Chen et al. analyzed the expression of LAT1 and LAT2 amino acid transporters in SGA infants and reported significantly lower levels compared to AGA and LGA cohorts (p < 0.01) (29). Furthermore, the study revealed a significant correlation between the expression of LAT1 and LAT2 and FBW in the total study population (p < 0.01) (29).

### 3.9 Activity of system L transporters in fetal growth restriction and small-for-gestational-age infants

With regard to FGR, Jansson et al. demonstrated that System L activity was significantly reduced in pregnancies with concomitant FGR, with alterations observed in both the syncytial MVM (-46%, p < 0.05) and BM (-38%, p < 0.05) (34).

No studies investigated the activity of System L in SGA infants.

### 3.10 Expression of system L transporters in fetal macrosomia and large-for-gestational-age infants

Only one study described System L expression in LGA infants (29). As mentioned earlier, Chen et al. observed a significantly higher expression of LAT1 and LAT2 amino acid transporters in LGA neonates as compared to SGA (both p < 0.01), whereas no significant differences between LGA and AGA were noted.

### 3.11 Activity of system L transporters in fetal macrosomia and large-for-gestational-age infants

Available studies examining the functionality of System L in cases of fetal overgrowth have yielded contradictory data. Jansson et al. reported significantly increased uptake of leucine into MVM vesicles among women with GDM delivering LGA infants as compared to controls (p < 0.05) (35). In contrast, Kuruvilla et al. found no significant difference in System L activity in placentas from macrosomic infants (38). While this study observed a substantial 49% reduction in System A activity in the macrosomic group, the System L maximal velocity (V_max_) and transporter affinity (K_m_) remained comparable to AGA infants, indicating that fetal macrosomia in this cohort was not driven by an upregulation of leucine transport capacity (38).

### 3.12 Expression of system L transporters in diabetes mellitus

Two studies analyzed the placental expression of LAT amino acid transporters in pregnancies complicated by DM (28, 41). The expression of LAT1 was evaluated in both studies, yielding mixed evidence (28, 41). While Castillo-Castrejon reported protein levels increased by 27% (p = 0.01) in T2DM, which correlated with FBW (r = 0.59, p = 0.04) and neonatal fat mass (r = 0.76, p = 0.06), Sakuragi et al. observed no significant variations regarding the per villus unit expression of LAT1 among women with different types of glucose intolerance [0.55 (0.51-0.61 vs. 0.58 (0.54-0.62), p = 0.20) (41). Of importance, when the per placental unit expression was assessed, LAT1 levels were significantly higher in the affected women (p < 0.001) (41). Investigations into the second System L isoform revealed that MVM LAT2 expression increased by 59% (p = 0.01) in T2DM patients, yet this upregulation showed no significant relationship with either FBW or neonatal adiposity (28). Lastly, the expression profile of System L transporters within the syncytiotrophoblast basal membrane demonstrated statistical parity (28).

### 3.13 Activity of system L transporters in diabetes mellitus

Two scholarly works focused on evaluating the functional activity of System L in the placenta during diabetic pregnancies (35, 38). Both research papers reported that the System L function is not significantly altered in diabetic subjects, provided that fetal macrosomia or LGA is not concurrently present. Findings from Jansson et al. suggest that System L-mediated leucine uptake deviates significantly from levels recorded in healthy individuals, exclusively in the GDM-LGA cohort (35). In addition, Kuruvilla et al. reported that in pregnancies affected by DM (T1DM, GDM, and impaired glucose tolerance) with AGA infants, System L activity in microvillous membrane vesicles was comparable to that of non-DM controls, further confirming that transporter function remains preserved in diabetic placentas when fetal growth is normal (38).

## 4 Discussion

To our knowledge, this is the first systematic review evaluating the activity and expression of System A and System L amino acid transporters in placentas from pregnancies affected by maternal DM and fetal growth disorders. Comprehensive analysis of 17 distinct studies facilitated the identification of two contrasting placental phenotypes. Pregnancies associated with FGR/SGA primarily manifest a reduction in the activity of System A and L, coupled with decreased protein expression of the constituent isoforms. In contrast, those involving DM and fetal macrosomia are characterized by an elevation in transporter functionality and protein levels. Although data regarding the expression of specific isoforms is not fully coherent, our findings confirm that placental transporter capacity adapts - or fails to adapt - to the maternal metabolic environment and fetal growth demands.

Reduced amino acid transfer represents a hallmark of FGR and SGA infants. All analyzed papers unanimously reported a significant reduction in the activity of placental System A and System L in the aforementioned growth disorders (19, 30–32, 34, 40). Although not so consistent at the protein level, this functional deficit appears to be driven by a reduction in transporter abundance at the syncytial plasma membranes, including decreased expression of SNAT and LAT isoforms (27, 29, 30, 33, 37). Collectively, these findings support the hypothesis that placentas from FGR/SGA-affected pregnancies are characterized by a reduced density of functional transporters, thereby limiting the substrate availability for the growing fetus. Importantly, this functional impairment may not be limited to the maternal interface but also affects the fetal-facing basal membrane, thus creating a ‘double bottleneck’ for nutrient transfer (34). This comprehensive reduction in transport capacity suggests that the placenta fails to meet the metabolic demands of the fetus, directly contributing to the phenotype observed in FGR, and possibly in SGA.

While the available evidence points toward general downregulation of amino acid transport, our review identified several important nuances regarding the impact of gestational age at diagnosis, associated severity of FGR and presence of maternal PE. It has been established that transporter expression and activity undergo unique changes in cases of severe and early-onset FGR, which are not observed in the milder or late-onset clinical variants (31, 37). These observations imply that the severity of the placental pathology during early gestation leads to a more substantial loss of transport efficiency, providing further evidence for a functional link between placental amino acid transport capacity and fetal development. Finally, contrary to what could be expected, the expression levels and functional capacity of SNAT and LAT transporters remained unaltered or even increased in gestations complicated by PE (19, 33, 37) This data supports the hypothesis that PE and isolated FGR represent distinct pathological entities, rather than manifestations of a single placental insufficiency spectrum. While the syncytiotrophoblast mounts a compensatory upregulation of System L amino acid transporters in response to the nutrient-limiting environments of both PE and FGR, the upstream regulatory mechanisms diverge significantly. Specifically, isolated FGR utilizes the mTOR signaling pathway to drive this adaptation, a response that is conspicuously absent in pregnancies with concomitant maternal PE (27).

In contrast to pregnancies affected by FGR, those complicated by fetal overgrowth are generally associated with an upregulated placental amino acid transfer, with maternal DM acting as an important modulatory factor. The findings of Kuruvilla et al. showing reduced System A and unaltered System L activity in cases of fetal macrosomia in pregnancies with concomitant DM stand as outliers against the broader literature (38). It is possible that certain characteristics of the aforementioned study population, including patients’ ethnicity, nutritional status, or clinical management of DM during gestation, in conjunction with a different methodological approach, contributed to observed discrepancies when contrasted with more recent reports. Importance of DM in modulating maternal-fetal amino acid transfer is further supported by results of Jansson et al., who demonstrated that System A activity is significantly increased in T1DM/GDM-complicated pregnancies (35). The observed upregulation appears to be driven by the diabetic environment itself, as transport activity was elevated in pregnancies with concomitant DM regardless of fetal size and remained unaltered in non-diabetic LGA infants.

Another critical insight emerging from this review is the distinction between “transporter density”, characterized as expression per milligram of protein, and “total organ capacity”, representing total expression per placental unit. Sakuragi et al. provided a key resolution to this conflicting expression data. They found that in women with different types of glucose intolerance (T2DM/GDM/overt DM), the density of SNAT1 protein per placental villus was significantly lower as compared to that in normoglycemic women (41). However, because these placentas were hyperplastic, the total placental expression of SNAT1 and LAT1 isoforms was significantly higher. The proposed hypothesis explains how a placenta with “inefficient” cells (e.g. with lower transporter density) can still result in a delivery of a macrosomic fetus: the sheer increase in placental mass compensates for the cellular defect, resulting in a net surplus of amino acid delivery.

Notably, the current literature review highlights a potential correlation between the expression and activity of placental amino acid transporters and neonatal body composition, extending beyond simple birth weight metrics. Specifically, Harrington et al. demonstrated that System A activity in SGA pregnancies correlates positively with neonatal skinfold thickness, serving as a functional proxy for fetal soft tissue accretion (32). Moreover, in pregnancies complicated by T2DM, Castillo-Castrejon et al. identified an association between the expression of LAT1 and neonatal fat mass (28). Such evidence implies that the upregulation of particular transporter isoforms within the placental barrier could be responsible for the differential growth of fetal tissues during gestation.

It must be acknowledged that the activity and expression of placental amino acid transporters are influenced by numerous confounding factors. For instance, the specific contribution of individual transporter isoforms can shift significantly depending on the gestational age at delivery (43). Additionally, maternal obesity and a higher BMI are inversely correlated with System A activity, likely driven by obesity-induced maternal hyperleptinemia and subsequent placental leptin resistance (44, 45). Ultimately, the implementation of a high-fat dietary regimen in animal model results in elevated SNAT2 gene and protein levels in a sex-specific manner, underscoring the significance of sexually dimorphic placental responses in the mediation of maternal-fetal nutrient exchange (44, 45).The presented systematic review has several key strengths. First, to our knowledge, it is the only study published so far that comprehensively synthesizes available data on both System A and System L amino acid transporter expression and activity across the full spectrum of fetal growth disorders (FGR/SGA and fetal macrosomia/LGA) and different types of DM. By integrating activity data with transporter expression levels, this review moves beyond simple analysis of protein abundance to provide a more functional view of the maternal-fetal nutrient flux. Another strength of this review is its transparent methodology, including adherence to PRISMA guidelines, a registered PROSPERO protocol, and a rigorous search conducted in four major databases. It is essential to highlight that collected data were stratified by specific fetal growth phenotypes, distinguishing between early- and late-onset FGR, and between diabetic and non-diabetic fetal overgrowth. Implementation of this approach allowed for the identification of distinct biological characteristics, which could be lost in a broader aggregate analysis. Finally, the inclusion of studies correlating transporter expression and activity with neonatal anthropometric parameters (e.g., FBW and skinfold thickness) provides a translational perspective on how placental function shapes fetal growth and body composition, linking these molecular mechanisms to the developmental programming of long-term metabolic health.

Nevertheless, it is imperative to address certain limitations. First, the available literature is relatively scarce, with only 17 studies meeting the inclusion criteria, many of which relied on small sample sizes. This limits the statistical power to perform a meta-analysis or draw definitive conclusions regarding the role of less-known transporter isoforms like SNAT5. Secondly, significant heterogeneity in study design, particularly in the diagnostic criteria used for FGR and SGA, ranging from simple birth weight percentiles to the inclusion of Doppler velocimetry, likely induced selection bias in some of the publications, and thus hindered comparative analysis across investigations. This inconsistency in definitions is of critical importance because, as previously noted, placental amino acid transport impairment may be severity-dependent and become evident only in strictly defined FGR phenotypes. Furthermore, a significant source of potential heterogeneity among the evaluated studies stems from the frequent lack of adjustment for confounding factors. Variables such as maternal BMI, gestational weight gain, and the degree of glycemic control are recognized as independent modulators of placental function, capable of modifying amino acid transporter capacity even in the absence of overt diabetes. As previously mentioned, gestational age at delivery, as well as fetal sex and associated placental dimorphism, further add to this variability. Considering that a substantial proportion of the included studies, particularly older investigations, did not routinely stratify their data by these variables or employ multivariate regression analyses to isolate them, separating the independent effects of fetal growth disorders from these overlapping pregnancy conditions remains a challenge when synthesizing the available literature. It should also be acknowledged that the majority of evaluated studies relied on term placental tissue, which represents the end-stage of gestation. Consequently, it remains difficult to distinguish whether the observed alterations in the expression and activity of transporters are a primary driver of aberrant growth or a secondary adaptation to the maternal metabolic milieu. Lastly, methodological differences in measuring transporter activity (e.g., vesicle preparation vs. villous fragments) and the variability in antibody validation across older studies may contribute to discrepancies in reported results on the System A and System L functionality and protein expression.

## 5 Conclusions

Despite the relatively limited number of studies available, the consistency of functional data allows for a clear characterization of two placental phenotypes: FGR is almost universally associated with downregulated amino-acid transport activity, whereas diabetes-induced fetal overgrowth represents a more complex picture of upregulation or compensatory maintenance. While the current literature, restricted largely to term placental tissue, limits our ability to determine causality versus adaptation, the strong alignment between transporter activity and neonatal anthropometric measurements, such as birth weight and adiposity, underscores the role of amino-acid transfer in the programming of fetal growth and long-term metabolic health. As a consequence, transplacental flux of amino acids represents an important target for future investigations regarding the developmental origins of metabolic health in the offspring.

## Data Availability

The original contributions presented in the study are included in the article/**Supplementary Material**. Further inquiries can be directed to the corresponding author.

## Supplementary material

Supplementary File S1 Full search strategy.
