# Supplementary material for: Placental expression and activity of system A and system L amino acid transporters in pregnancies complicated by diabetes mellitus and fetal growth disorders: a systematic review
Source: Front Endocrinol (Lausanne). 2026 Jul 13;17:1883051. doi: 10.3389/fendo.2026.1883051 (PMC13402201; doi:10.3389/fendo.2026.1883051)
Supplement: Supplementary File S1 — Full search strategy. [file DataSheet1.pdf]

Embase:

('amino acid transporter'/exp OR 'amino acid transporter' OR 'amino acid transport system' OR 'system a' OR 'system l' OR snat OR snat1 OR snat2 OR snat4 OR slc38a1 OR slc38a2 OR slc38a4 OR lat OR lat1 OR lat2 OR slc7a5 OR slc7a8) AND ('placenta'/exp OR placenta OR placental OR 'chorionic villi' OR trophoblast OR syncytiotrophoblast) AND ('fetal growth retardation'/exp OR 'fetal growth restriction' OR fgr OR 'small for gestational age' OR sga OR 'large for gestational age' OR lga OR macrosomia OR 'fetal macrosomia') AND ('diabetes mellitus'/exp OR 'diabetes mellitus' OR 'gestational diabetes' OR gdm OR 'type 1 diabetes' OR t1dm OR 'type 2 diabetes' OR t2dm) AND (expression OR mrna OR transcript OR qpcr OR protein OR immunohistochem* OR western OR uptake OR transport OR activity OR functional) NOT ([animals]/lim NOT [humans]/lim)

Scopus:

(TITLE-ABS-KEY ("amino acid transporter" OR "amino acid transport system" OR "system a" OR "system l" ORsnat OR snat1 OR snat2 OR snat4 OR slc38a1 OR slc38a2 OR slc38a4 OR lat OR lat1 OR lat2 OR slc7a5 OR slc7a8)AND TITLE-ABS-KEY (placenta OR placental OR "chorionic villi" OR trophoblast OR syncytiotrophoblast) ANDTITLE-ABS-KEY ("fetal growth restriction" OR fgr OR "small for gestational age" OR sga OR "large for gestational age" OR lga OR macrosomia OR "fetal macrosomia") AND TITLE-ABS-KEY ("diabetes mellitus" OR"gestational diabetes" OR gdm OR "type 1 diabetes" OR t1dm OR "type 2 diabetes" OR t2dm) AND TITLE-ABS-KEY (expression OR mrna OR transcript OR qpcr OR protein OR immunohistochem * OR western OR uptake ORtransport OR activity OR functional))

PUBMED:
(

amino acid transport[tiab]

OR amino acid transporter[tiab]

OR "amino acid transport system"[tiab]

OR SNAT[tiab]

OR SNAT1[tiab]

OR SNAT2[tiab]

```
  OR SNAT4[tiab]
  OR SLC38A1[tiab]
  OR SLC38A2[tiab]
  OR SLC38A4[tiab]
  OR LAT[tiab]
  OR LAT1[tiab]
  OR LAT2[tiab]
  OR SLC7A5[tiab]
  OR SLC7A8[tiab]
  OR "system A"[tiab]
  OR "system L"[tiab]
)
AND
(
  placenta[tiab]
  OR placental[tiab]
  OR trophoblast[tiab]
  OR "chorionic villi"[tiab]
  OR syncytiotrophoblast[tiab]
  OR Placenta[MeSH Terms]
)
AND
(
  (
    "Fetal Growth Retardation"[MeSH Terms]
    OR "Infant, Small for Gestational Age"[MeSH Terms]
    OR "fetal growth restriction"[tiab]
    OR FGR[tiab]
```

```
    OR "growth restriction"[tiab]
    OR "small for gestational age"[tiab]
    OR SGA[tiab]
    OR "large for gestational age"[tiab]
    OR LGA[tiab]
    OR macrosomia[tiab]
    OR "fetal macrosomia"[tiab]
  )
  OR
  (
    "Diabetes Mellitus"[MeSH Terms]
    OR "Diabetes Mellitus, Type 1"[MeSH Terms]
    OR "Diabetes Mellitus, Type 2"[MeSH Terms]
    OR "Diabetes, Gestational"[MeSH Terms]
    OR "diabetes mellitus"[tiab]
    OR "gestational diabetes"[tiab]
    OR "type 1 diabetes"[tiab]
    OR "type 2 diabetes"[tiab]
    OR GDM[tiab]
    OR T1DM[tiab]
    OR T2DM[tiab]
  )
)
AND
(
  expression[tiab]
  OR activity[tiab]
  OR transport[tiab]
```

  OR functional[tiab]

)

NOT

(

  animals[MeSH Terms]

  NOT humans[MeSH Terms]

)

Web of science:

("amino acid transport" OR "amino acid transporter" OR "amino acid transport system" OR SNAT OR SNAT1 OR SNAT2 OR SNAT4 OR SLC38A1 OR SLC38A2 OR SLC38A4 OR LAT1 OR LAT2 OR SLC7A5 OR SLC7A8 OR "system A" OR "system L") AND (placenta OR placental OR trophoblast OR "chorionic villi" OR syncytiotrophoblast) AND ("fetal growth restriction" OR FGR OR "small for gestational age" OR SGA OR "intrauterine growth restriction" OR IUGR OR "large for gestational age" OR LGA OR macrosomia OR "fetal macrosomia" OR "diabetes mellitus" OR "gestational diabetes" OR GDM OR "type 1 diabetes" OR T1DM OR "type 2 diabetes" OR T2DM) AND (expression OR activity OR transport OR functional) NOT (mouse OR mice OR rat OR rats OR murine OR rodent* OR porcine OR pig OR swine OR bovine OR cow OR canine OR dog)

(("amino acid transport" OR "amino acid transporter*" OR "amino acid transport system" OR SNAT1 OR SNAT2 OR SNAT4 OR SLC38A1 OR SLC38A2 OR SLC38A4 OR LAT1 OR LAT2 OR SLC7A5 OR SLC7A8 OR "system A" OR "system L")

AND (placenta OR placental OR trophoblast OR "chorionic villi" OR syncytiotrophoblast)

AND ("fetal growth restriction" OR FGR OR "small for gestational age" OR SGA OR "large for gestational age" OR LGA OR macrosomia OR "fetal macrosomia" OR "intrauterine growth restriction" OR IUGR OR "diabetes mellitus" OR "gestational diabetes" OR GDM OR "type 1 diabetes" OR T1DM OR "type 2 diabetes" OR T2DM)

AND (expression OR activity OR transport OR "functional activity" OR "transporter expression"))

NOT (mouse OR mice OR rat OR rats OR murine OR rodent* OR porcine OR pig OR swine OR bovine OR cow OR sheep OR ovine OR canine OR dog)
